# Methylation Landscape of Human Breast Cancer Cells in Response to Dietary Compound Resveratrol

**DOI:** 10.1371/journal.pone.0157866

**Published:** 2016-06-29

**Authors:** Rubiceli Medina-Aguilar, Carlos Pérez-Plasencia, Laurence A. Marchat, Patricio Gariglio, Jaime García Mena, Sergio Rodríguez Cuevas, Erika Ruíz-García, Horacio Astudillo-de la Vega, Jennifer Hernández Juárez, Ali Flores-Pérez, César López-Camarillo

**Affiliations:** 1 Departamento de Genética y Biología Molecular, CINVESTAV-IPN, Ciudad de México, México; 2 Laboratorio de Genómica Funcional, Unidad de Biomedicina, FES-Iztacala UNAM, Tlalnepantla, Estado de México, México; 3 Programa en Biomedicina Molecular y Red de Biotecnología, Escuela Nacional de Medicina y Homeopatía, Instituto Politécnico Nacional, Ciudad de México, México; 4 Instituto de Enfermedades de la Mama, FUCAM, Ciudad de México, México; 5 Laboratorio de Medicina Translacional, Instituto Nacional de Cancerología, Ciudad de México, México; 6 Laboratorio de Investigación en Cáncer y Terapia Celular, Hospital de Oncología, Centro Médico Siglo XXI, Ciudad de México, México; 7 Posgrado en Ciencias Genómicas, Universidad Autónoma de la Ciudad de México, Ciudad de México, México; University of Navarra, SPAIN

## Abstract

Aberrant DNA methylation is a frequent epigenetic alteration in cancer cells that has emerged as a pivotal mechanism for tumorigenesis. Accordingly, novel therapies targeting the epigenome are being explored with the aim to restore normal DNA methylation patterns on oncogenes and tumor suppressor genes. A limited number of studies indicate that dietary compound resveratrol modulates DNA methylation of several cancer-related genes; however a complete view of changes in methylome by resveratrol has not been reported yet. In this study we performed a genome-wide survey of DNA methylation signatures in triple negative breast cancer cells exposed to resveratrol. Our data showed that resveratrol treatment for 24 h and 48 h decreased gene promoter hypermethylation and increased DNA hypomethylation. Of 2476 hypermethylated genes in control cells, 1,459 and 1,547 were differentially hypomethylated after 24 h and 48 h, respectively. Remarkably, resveratrol did not induce widespread non-specific DNA hyper- or hypomethylation as changes in methylation were found in only 12.5% of 27,728 CpG *loci*. Moreover, resveratrol restores the hypomethylated and hypermethylated status of key tumor suppressor genes and oncogenes, respectively. Importantly, the integrative analysis of methylome and transcriptome profiles in response to resveratrol showed that methylation alterations were concordant with changes in mRNA expression. Our findings reveal for the first time the impact of resveratrol on the methylome of breast cancer cells and identify novel potential targets for epigenetic therapy. We propose that resveratrol may be considered as a dietary epidrug as it may exert its anti-tumor activities by modifying the methylation status of cancer -related genes which deserves further *in vivo* characterization.

## Introduction

Epigenetic aberrations and specific alterations in DNA methylation patterns resulting in altered gene expression programs may greatly contribute to tumorigenesis [1]. Global hypomethylation and site-specific hypermethylation of gene promoters occur in many tumors including breast, colon, lung and prostate cancer [2]. Hypomethylation of CpG islands can result in genome instability, reactivation of transposons, and upregulation of proto-oncogenes [3], whilst promoter hypermethylation may suppress the transcription of tumor suppressor genes, including genes involved in DNA repair, detoxification, apoptosis, cell cycle, cell proliferation, metastasis and angiogenesis [4]. In contrast to genetic modifications, epigenetic deregulation of cancer cells is potentially reversible and restoration of normal DNA methylation marks has been established as a promising strategy in cancer therapeutics. Accordingly, novel therapies targeting the epigenome are being explored with the aim to restore normal DNA methylation patterns on oncogenes and tumor suppressor genes. In this context, increasing experimental evidence suggest that dietary compounds may exert health benefits through the modulation of the epigenetic status of cells during the lifespan [5]. Many phytochemicals found in vegetables and plants have potent antioxidant and antitumor activities with low toxicity. These nutraceuticals may alter the epigenetic marks involved in the early steps of carcinogenesis, such as global DNA hypomethylation, tumor suppressor gene promoter hypermethylation and modifications of the histones code [6]. Therefore the search and discovery of novel dietary epigenetic modulators and their clinical application in patients is an emerging therapeutic strategy against human cancers.

Resveratrol (3, 5, 4′-trihydroxy-trans-stilbene) polyphenol is a phytoalexin found in grapes, berries, peanuts, chocolate, red wine, herbs and plants. This nutraceutical exhibits antitumor activities in diverse types of human cancers. Numerous studies, using both *in vitro* and *in vivo* model systems, have illustrated that resveratrol can modulate specific signaling pathways associated with cell growth and division, apoptosis, angiogenesis, invasion, and metastasis in cancer [7]. Interestingly, a limited number of studies suggest that dietary resveratrol may exert its chemopreventive and therapeutic effects in cancer cells through epigenetic mechanisms [8–11]. However a complete view of methylation changes in epigenome after resveratrol treatment has not been reported yet in cancer. In this study we performed a genome-wide survey of DNA methylation in triple-negative MDA-MB-231 breast cancer cells exposed to resveratrol using the array-based profiling of reference-independent methylation status (aPRIMES) followed by whole-genome hybridization using human DNA methylation promoter microarrays. Our data indicate that resveratrol reverses DNA methylation alterations of specific genes and pathways in breast cancer cells. In addition integrative analysis of DNA methylation and gene expression at different times of resveratrol exposure showed that changes in DNA methylation were associated to corresponding changes in mRNA expression in a set of cancer-related genes. The implications that these findings might have in breast cancer chemoprevention and therapy are discussed.

## Materials and Methods

### Cell cultures and reagents

MDA-MB-231 breast cancer cell line was obtained from the American Type Culture Collection. Cells were maintained in Dulbecco’s modified Eagle’s minimal essential medium (DMEM), supplemented with 10% fetal bovine serum and antibiotics (100 U/ml penicillin and 100 U/ml streptomycin) at 37˚C in a humidified atmosphere of 5% CO2. Resveratrol was purchased from Sigma Aldrich (St. Louis, MO, USA), and dissolved at 80 mmol/l concentration, and diluted with DMEM to 100 μM working concentration.

### Genome-wide analysis of DNA methylation by array-PRIMES (aPRIMES)

The extraction of high molecular weight DNA of the cells MDA-MB-231 untreated and treated with resveratrol was extracted using the DNeasy Kit (Qiagen, Germany) according to the manufacturer’s instructions. To determine the methylated and unmethylated DNA regions in the promoters of genes, we used Array-PRIMES method (aPRIMES) as described previously (12). aPRIMES is based on the differential restriction and competitive hybridization of DNA by methylation-specific and methylation-sensitive restriction enzymes, respectively. Briefly, 500 ng genomic DNA was restricted to completion with 10 U MseI for 3 h in a final volume of 10 ml in the buffer provided by the supplier (New England Biolabs, Beverly, USA). Heat inactivation was carried out at 65°C for 20 min. MseI fragments were then subjected to linker-mediated PCR as essentially described (Klein, et al., 1999). Briefly, 1 ml each of 100 mM stock solution (MWG, Ebersberg, Germany) ddMse11 (50 -TAA CTGACAG-30) and Lib1 (50 -AGTGGGATTCCTGCTG TCAGT-30) were annealed in 1 ml One-Phor-All-Buffer and 3 ml ddH2O. Annealing was started at a temperature of 65°C and was shifted down to 15°C with a ramp of 1°C /min. At 15°C, 10 ml MseI fragments, 2 ml of ATP (10 mM) and 2 ml T4-DNA ligase (10 U; Roche, Grenzach-Wyhlen, Germany) were added, and primers and DNA fragments were ligated overnight. Half of the resulting ligated MseI fragments were digested with the restriction enzyme McrBC (New England Biolabs, Beverly, MA, USA) for 8 h. The other half of the MseI fragments was digested with the two methylation-sensitive endonucleases HpaII (New England Biolabs; recognition site CCGG, 3 h, 37°C) and BstUI (New England Biolabs; recognition site CGCG, 3 h, 60°C) according to the recommendations of the supplier. Digested DNA fragments were then treated with 1 ml proteinase K (Invitrogen, Karlsruhe, Germany) for 1 h at 37°C with subsequent heat inactivation at 80°C for 10 min. For the following amplification step, 10 ml consisting of 2 ml 10 Expand Long Template buffer 1 (Boehringer, Mannheim, Germany), 1 ml dNTPs (10 mM), 1 ml Lib1 primer (50 -TAACTAGCATGC-30), 1 ml expand long template DNA polymerase mixture (Boehringer, Mannheim, Germany) and 5 ml H2O were added to 20 ml reaction volume. A MWG thermocycler was programmed to 72°C for 3 min, followed by 20 cycle loops at 94°C (30 s), 62°C (30 s) and 72°C (90 s). Final elongation was carried out at 72°C for 10 min. PCR products were recovered by ethanol precipitation. DNA was eluted in 30 ml 0.1 TE, pH 8.0.

### DNA microarrays

For DNA methylation analysis we used Nimblegen HG18 Refseq Promoter 3x720K array. The array contained 720,000 probes of 50–75 bp in length with a median probe spacing of 104 bp, covering 30,848 transcripts, 22,532 promoters, and 27,728 CpG islands. 1.5 μg of experimental (IP) and control (input) DNA was directly labeled by Klenow random priming with Cy3 and Cy5 nonamers with NimbleGen Dual-color DNA Labeling Kit following manufacturer’s user’s guide, and the labeled DNA was precipitated with 1 volume isopropanol. Hybridization mix including 15 mg of labeled DNA was prepared using NimbleGen Hybridization Kit. Arrays were hybridized in NimbleGen Hybridization System 4 Station for 18 h at 42°C, and then washed in 1X Wash solution I, II and III. Hybridization buffers and washes were completed using manufacturer’s protocols. Arrays were scanned on a NimbleGen MS 200 Scanner per manufacturer’s protocol. DNA methylation analysis raw data was normalized and differential intensity of each probe compared with input control was calculated using the NimbleGen software DEVA. Average fold change (IP versus input) each 50 bp bin for a range of 2.44 kb upstream and 610 bp downstream window from RefSeq transcription start sites (TSS). Functional annotation of target genes based on Gene Ontology was performed using DAVID Software (Database for Annotation, Visualization and Integrated Discovery).

### Microarray data processing

Identification of probes with significant scaled log2 ratio was performed by DEVA software v. 1.2.1. (Roche NimbleGen). The signal intensity ratios, were generated by subtracting the log transformed IP channel intensities from the log transformed Input channel intensities. The ratios were centered on a per sample basis by the Tukey biweight function. Probes with significant scaled log2 ratio were identified by DEVA software using default parameters as provided by the manufacturer. An algorithm derived from a modified Kolmogorov–Smirnov test was used to predict enriched regions representing methylated CpG islands across multiple adjacent probes in sliding-windows 100 base pairs in length. Differentially enriched regions of experimental vs control DNA were identified based on number and coverage of bound probes to methylated fragments. The mean log-ratio of samples was integrated across the enriched regions. The methylation peaks were mapped to features using DEVA software. Regions showing enrichment at 4 or more consecutive loci were integrated together to form a single “peak”. Clusters of enriched regions separated by more than 500 base pairs were integrated as separate peaks, which reflected the probability of methylation for the designated peak and/or gene at a p-value of less than 0.01.

### Gene Ontology (GO) and pathways analysis

The Database for Annotation, Visualization and Integrated Discovery (DAVID version 6.7) software (http://david.abcc.ncifcrf.gov/) was used to perform GO and PATHWAY analysis for regulatory network. DAVID provides a comprehensive set of functional annotation tools to understand biological meaning behind large lists of genes.

### Statistical analysis

A two way ANOVA was performed to identify differentially methylated genes. Only genes with statistically significant differences in DNA methylation levels (p-value <0.05) were included. Statistical analysis was performed using by SPSS (SPSS, Chicago, IL, USA) and Microsoft Excel software.

## Results

### Genome-wide identification of DNA methylation changes in breast cancer cells treated with resveratrol

**Fig 1:**
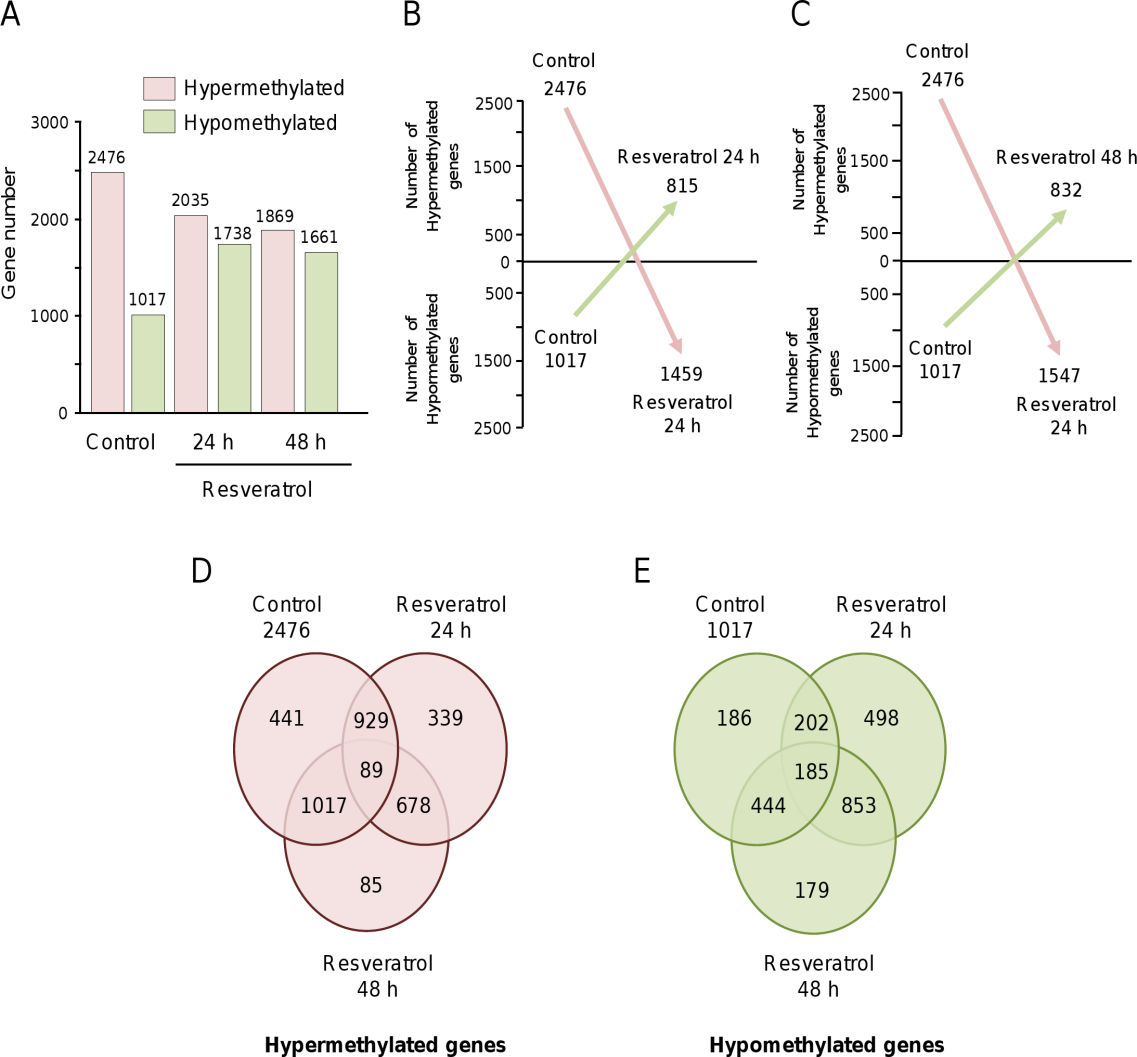
Epigenetically modulated genes in MDA-MB-231 breast cancer cells exposed to resveratrol. (A) Gene numbers with a significant change in DNA methylation (Δβ-value ≥1.5) after resveratrol treatment for 24 h and 48 h. (B-C) Schematic diagrams showing the changes in number of hypermethylated to hypomethylated genes (black arrows) and vice versa (grey arrows) after treatment with resveratrol at 24 h (B) and 48 h (C) in comparison to non-treated MDA-MB-231 cells. Venn diagrams summarizing the number of hypermethylated (D) and hypomethylated (D) genes in resveratrol treated cells with respect to control. The intersection among three circles indicates the hypermethylated genes and hypomethylated genes shared in the control and resveratrol treated cells.

To evaluate the epigenetic changes of triple-negative MDA-MB-231 breast cancer cells treated with resveratrol (100 μM) for 24 h and 48 h, we performed a genome-wide DNA methylation analysis. The methylated DNA regions were enriched using the array-based profiling of reference-independent methylation status (aPRIMES) method which is based on the differential restriction of DNA by methylation-specific and methylation-sensitive restriction enzymes [12], followed by whole-genome hybridization of human DNA methylation 3x720 K CpG Island Plus RefSeq promoter array HG18 CpG (NimbleGen) that covers 22,532 gene promoters and 27,728 annotated CpG islands. To highlight the changes in DNA methylation status and to allow downstream processing and analyses, we used the DNA methylation workflow as implemented in the DEVA software version 1.2.1 by taking the default parameters. The DNA regions that displayed a peak score between >0.9 and <0.9 (Δβ-value of ≥1) were considered as hyper- and hypomethylated, respectively. We quantified the number of loci that underwent a change from a baseline methylation level in 0.9 methylation peak in order to include as many as possible changes in probes that underwent alterations in DNA methylation. Our data indicate that thousands of promoter genes were significantly hypo- or hypermethylated after resveratrol incubation in comparison to non-treated cells (S1, S2 and S3 Tables). Resveratrol treatment for 24 h and 48 h induced a significant decrease in DNA hypermethylation of promoter genes accompanied with an increase in hypomethylation. Moreover, we found that this polyphenol induced specific and limited changes in DNA methylation, instead of pleiotropic effects, because the alterations were only detected in 12.5% of the 27,728 CpG *loci* studied here. A total of 2,476 hypermethylated and 1,017 hypomethylated gene promoters were identified in control MDA-MB-231 cells without treatment (Fig 1A). After 24 h resveratrol intervention, 2035 and 1,738 genes were hyper- and hypomethylated, respectively, whereas at 48 h treatment 1,869 and 1,661 genes showed low and high methylation levels, respectively (Fig 1A). Then we asked whether changes in methylation signatures detected by aPRIMES approach were differential after two times of resveratrol treatment. Of 2,476 hypermethylated gene promoters, 1,459 (58.9%) and 1,547 (62.4%) *loci* were differentially hypomethylated after 24 h and 48 h treatment, respectively, in comparison to non-treated cells (Fig 1B and 1C). Moreover, after 24 h and 48 h incubation with the polyphenol, 815 (80.0%) and 832 (81.8%) of gene promoters were differentially hypermethylated, respectively, in comparison with 2,476 hypomethylated gene promoters in control cells (Fig 1B and 1C*)*. Venn diagram showed that of total 2476 hypermethylated genes in control cells, 1018 and 1106 genes remained hypermethylated after 24 h and 48 h treatment, respectively (Fig 1D). Likewise, of total 1017 hypomethylated genes in control cells, 386 and 629 genes remained hypomethylated after 24 h and 48 h treatment, respectively (Fig 1E).

**Fig 2:**
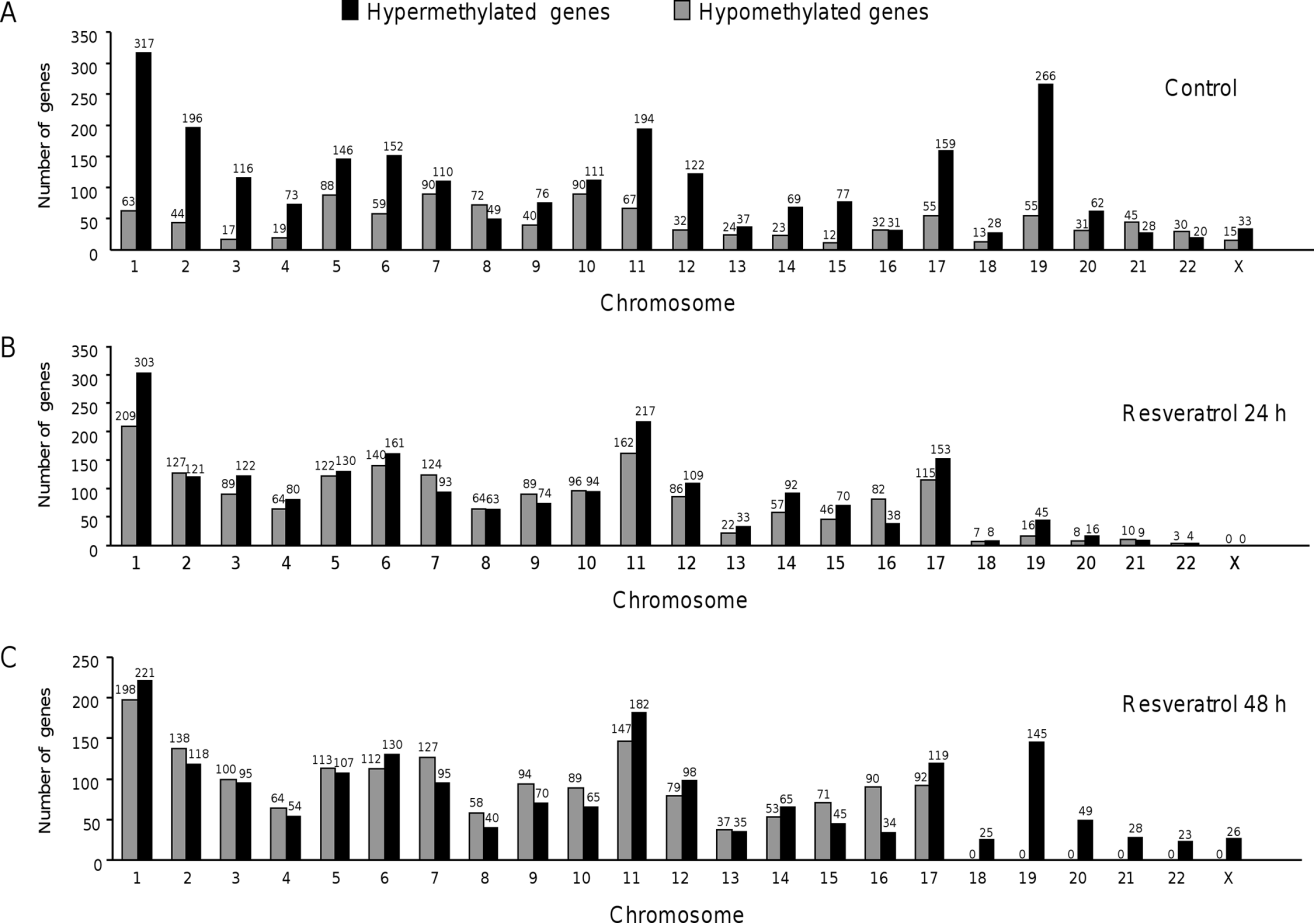
Distribution of hypermethylated and hypomethylated gene promoters along each chromosome. (A) Non-treated MDA-MB-231 control cells; treated with resveratrol (100 μM) for 24 h (B) and 48 h (C). Numbers up the bars indicate the amount of genes according to their position in chromosomes.

Then we sought to classify the changes in DNA methylated regions identified by aPRIMES according to its chromosomal location. Our genomic analysis showed the presence of intensive hypermethylation in chromosomes 1, 2, 5, 6, 11, 17 and 19 in MDA-MB-231 control cells, while a major number of hypomethylated genes was mainly distributed in chromosomes 1, 5, 7, 8, 10 and 11 (Fig 2A). After 24 h resveratrol treatment, we observed a major hypermethylation in chromosomes 1, 6, 11 and 17 at 24 h and hypomethylation in chromosomes 1 to 12 and 17 (Fig 2B). Likewise in resveratrol treated cells at 48 h we found hypermethylated gene promoters mainly in chromosomes 1, 6, 11 and 19, while the major number of hypomethylated gene promoters was found in chromosomes 1 to 17 (Fig 2C).

**Fig 3:**
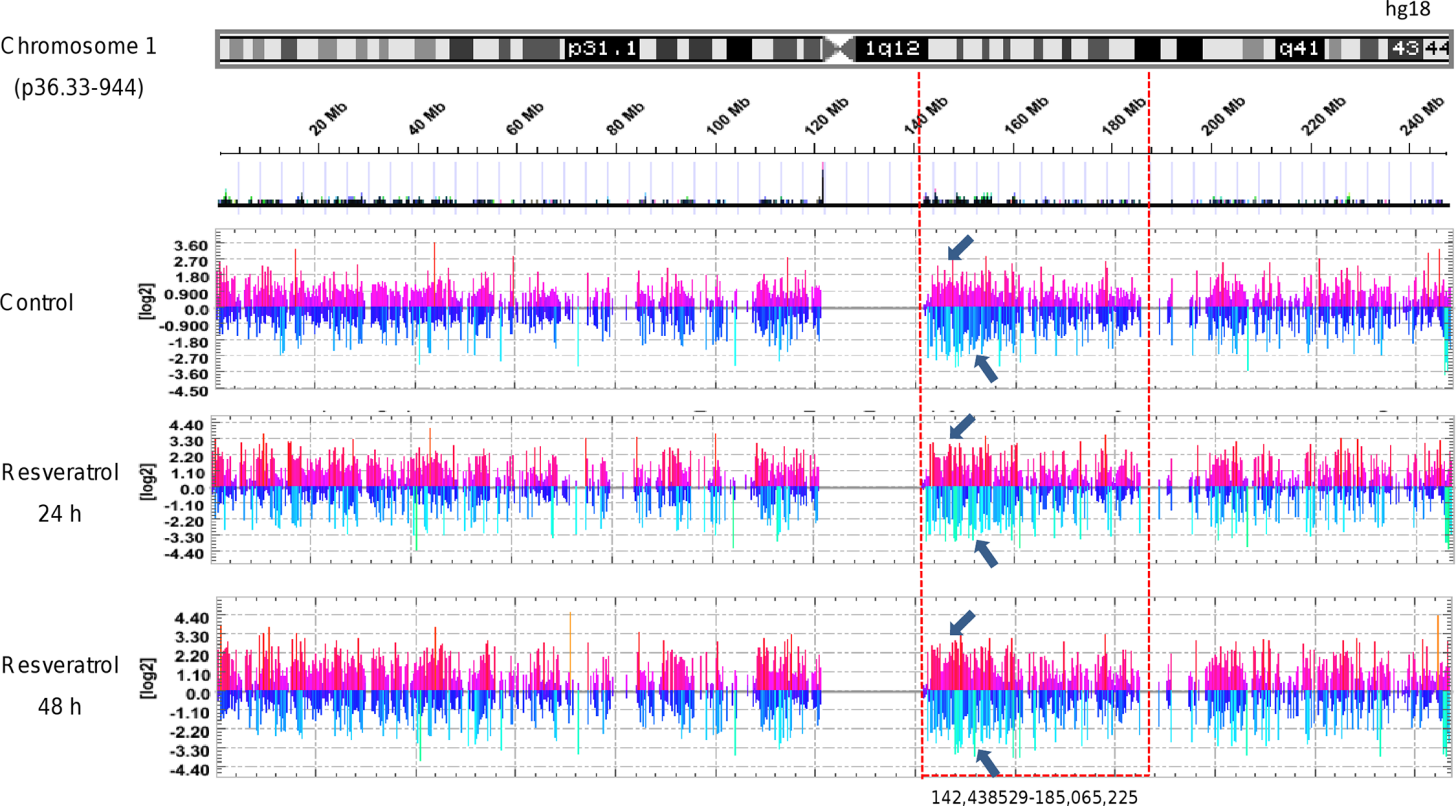
DNA methylation patterns along chromosome 1 in MDA-MB-231 breast cancer cells treated with resveratrol. (A) Schematic representation of the chromosome 1 as displayed in the UCSC genome browser together with the RefSeq genes. (B) Methylation signals (log2 of probe intensity 0 to ±4.0) around transcription start sites (-3200 to 800 bp) for all the genes ordered according to their position in the chromosome 1. Hypermethylated genes are marked with grey bars up to threshold; and hypomethylated genes with light grey and black bars down the threshold in control and resveratrol treated cells. Black dotted box denotes the genomic region 140–180 Mb of chromosome 1 and arrows indicate specific regions with significant changes in methylation after resveratrol treatment at both 24 h and 48 h.

To better understand the changes in DNA methylation in specific genomic regions we analyzed the whole 27,728 *loci* throughout the 23 chromosomes. A representative map of the annotation window representing the methylation signals (log2 of probe intensity 0 to ±4.0) in chromosome 1 is shown in Fig 3. In this particular example, we found a significant increase of the hypomethylation signals in the genomic region 140–180 Mb of chromosome 1 after 24 h and 48 h treatments relative to control (Fig 3). These data indicate that resveratrol was able to differentially alter the methylation status at specific chromosomes and particular CpG *loci* in a time-dependent manner in breast cancer cells.

**Fig 4:**
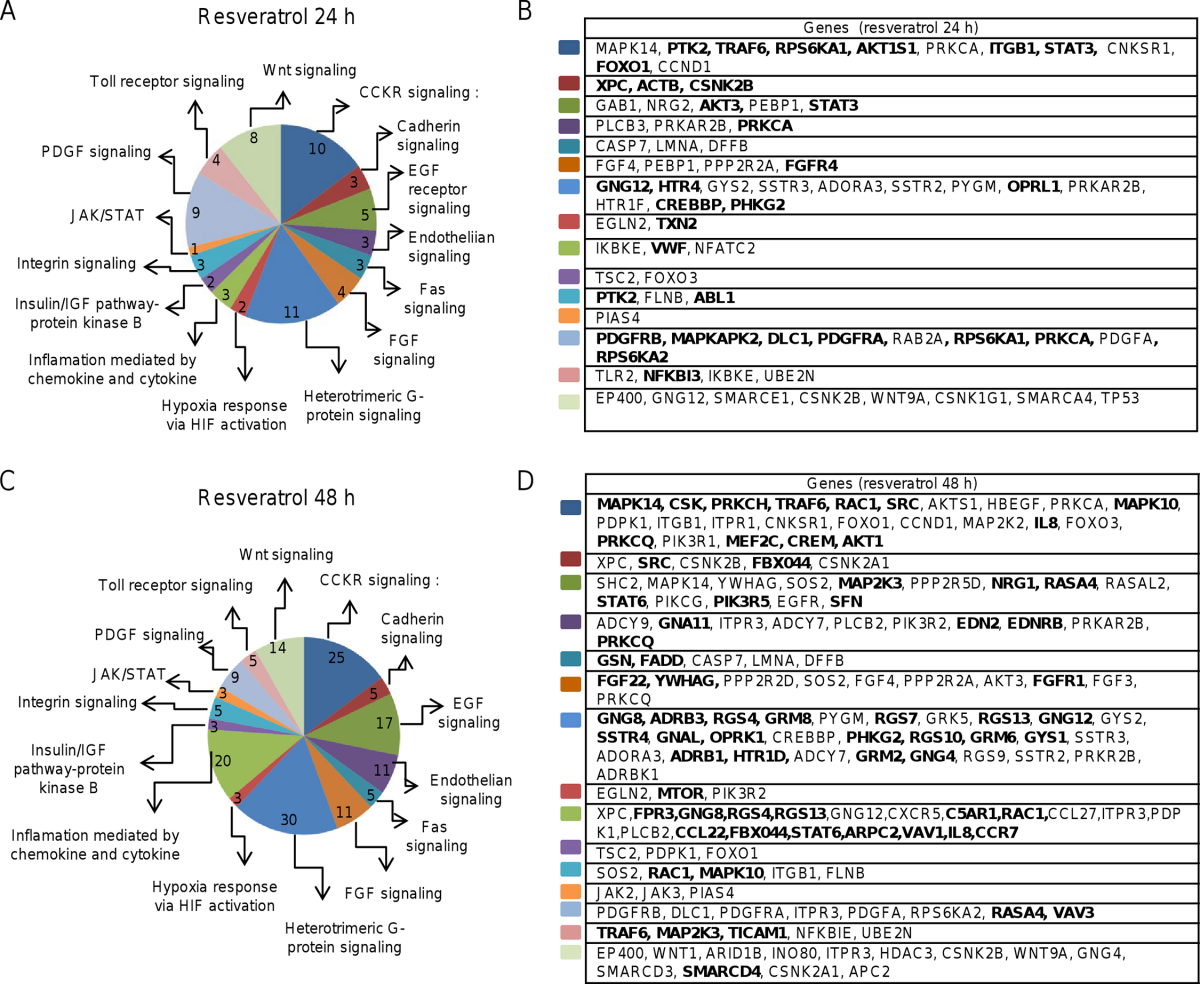
Analysis of cellular pathways. Signaling pathways epigenetically modulated by resveratrol treatment for 24 (A) and 48 (B) hours as predicted by Panther software. Right boxes denote the oncogenes (thin) and tumor suppressor genes (bold) involved in the signaling pathways affected by resveratrol.

As gene expression can be modulated by DNA methylation mechanisms, we asked about the impact of epigenetic changes induced by resveratrol in cellular pathways relevant to breast cancer. We performed a bioinformatics analysis using Panther in order to identify the biological pathways potentially affected by genes whose DNA methylation status was altered by resveratrol. Results showed that the majority of the gene promoters differentially methylated are involved in a wide variety of biological functions such as cell cycle, immune system, DNA repair, GPCR signaling, chromatin organization, cellular responses to stress, apoptosis, and glucose metabolism (Tables 1 and 2). A number of genes participates in cellular pathways involved in cancer development such as CCKR signaling (PTK2, TRAF6, RPS6KA1, AKT1S1, ITGB1, STAT3, FOXO1, MAPK14, CSK, PRKCH, RAC1, SRC, MAPK10, IL8, PRKCQ, MEF2C, CREM, AKT1), Wnt signaling (SMARCD4), PDGF signaling (PDGFRB, MAPKAPK2, DLC1, PDGFRA, RPS6KA1, PRKCA, RPS6KA2, RASA4, VAV3), Toll receptor signaling (TRAF6, MAP2K3, TICAM1), Jak-STAT signaling (JAK2, JAK3, PIAS4) and inflammation mediated by chemokine and cytokine FPR3, GNG8, RGS4, RGS13, C5AR1, RAC1, CCL22, FBX044, STAT6, ARPC2, VAV1, IL8, CCR7) among others (Fig 4). In addition, the hypermethylation of tumor suppressor genes (WIF1, SOX17, ADAMTSL2, SLIT3, GATA5, and WNT9A) and hypomethylation of oncogenes (MSH2, MSH3, CHK1, and CHK2) identified in this study have already been previously reported in colon, prostate, lung and breast tumors [13–16].

**Table 1:** Cellular pathways and genes with differential DNA methylation in MDA-MB-231 cells at 24 h after treatment with resveratrol.

Pathway name	Number of genes	Hypermethylated (bold letters) and hypomethylated genes
**Cell cycle**	112	**DKC1, AURKA, HSPA2, RCC2, HIST1H2BK, MAD1L1, TCP10, TUBB6, PRKCA,RRP1, HIST1H3A, CSNK2B, SGOL1, TUBA3C, HIST1H3D, ANKLE2, TP53, CCNB1, NUP98, DLC1, RAD17, HSP90AA1, CENPN, UBE2E2, CENPO, PSMA7, PSME3, PSMB10, NHP2, RUVBL2, APEX1, RAE1, HIST1H4F, PPP2R3B, CENPP, UBE2N, REC8, ORC1L, E2F1,** TUBA1C, PSMB9, SYCP1, HIST1H2BI, TEX12, PRKAR2B, POLE, TUBA3E, SKP1, PPP2R2A, PIAS4, NCAPH, HIST1H3E, TUBA3D, UBC, INCENP, NUP210, MSH5, NUDC, RAB2A, FKBP6, BUB3, CCND1.
**Immune System**	264	**DLG4, PYCARD, RPS6KA1, RPS6KA2, ITGB1, IL1B, SPTBN5, ASB4, GH1, GH2, HLADOB, NLRP12, EIF4G3, MRC2, EIF4G1, EIF4G2, TRAF6, CSHL1, TRAF2, EGR1, RAET1G, NFKBIE, ASB2, DUSP10, UBE2K, AKT1S1, SEC24C, IFITM2, TRAF7, RNF144B, IL17RD, LGALS3, CANX, NLRX1, RASGRF2, CD180, SEC61G, HLADMA, CARD11, MX1UBA1, FOXO1, ANGPT1, PDGFRB, PDGFRA, TNFRSF1B, ATP6V0A2, PIK3AP1, TEC,**, MAPK14, KEAP1, PDGFA, GZMM, C1S, ZAP70, GHR, KIR3DL2, TLR2, ACTR3, FGF4, IFITM1, GAB1, IL6R, IKBKE, HERC5, UNC93B1, LIF, CD200R1, IL27RA, CCL17, LILRA2, NFATC2, TRIM14, FLNB, TRIM17, TCEB2, IRF4, SPTBN1, CTSL3, RAB7A, MBL2, RNF216, TRIM11, CD226, FOXO3, POLR3K, TRIM68.
**DNA Repair**	61	**GTF2H4, HIST1H2BK, RRP1, XRCC5, XRCC4, TP53, PPP4R2, RAD17, GPS1, KDM4B, KDM4A, APEX1, HIST1H4F, UBE2N, FAN1, FAM175A, EYA3, ATRIP, NEIL2, NEIL3, NTHL1, RAD50, WHSC1, POLM, USP10, UBE2I, XPC, KIAA1530, XPA, HERC2, PAXIP1, REV3L, CUL4B, NFRKB, TFPT, KIAA0146, ALKBH3, CUL4A, RTEL1, ABL1, C19orf40, BACH1, ACTB, CDS1, SUMO3, INO80B, DCLRE1A, ACTR5, FANCI, CSNK1G1.** ERCC6, UBC, RAD51L3, C1orf86, COPS3, DDB2, ERCC2, HIST1H2BI, POLR2J, POLE, PNKP, PIAS4.
**Signaling by GPCR**	230	**GRIN2C, DGKZ, DLG4, GRIN2D, GPR39, PRKCA, ABR, TAS1R3, RPS6KA1, ADORA3, RPS6KA2, GHRL, HTR4, SSTR2, DGKI, SHH, DGKD, NPB, GOLT1A, PLXNB1, SPTBN5, CCR10, OPRL1, PSME3, PSMB10, FGFR4, GNG12, MGLL, NPSR1, OR4F5, OR4F4, VWF,.** PSMB9, PEBP1, SLA2, MLN, PROKR1, PLCB3, CXCL12, CNKSR1, GPR37, TRPC7, P2RY2, P2RY4, SST, HTR1F, SSTR3, NRG2, RGR, TAS2R4, AGT, WNT9A, ARHGEF16, ARHGEF17.
**Chromatin organization**	52	**DR1, BRD1, CHD3, CHD4, HIST1H2BK, KDM3A, SMARCA4, MLL, TRRAP, SMARCE1, KDM5B, KDM5C, HIST1H30A, MLL2, HIST1H3D, MLL3, PADI2, NCOR2, PHF8, EHMT1, MTA1, PRDM16, DNMT3A, PADI1, YEATS4, CSRP2BP, KDM4B, KDM4A, CREBBP, RUVBL2, EP400, SUPT3H, HIST1H4F, SETD1B, DOT1L, ACTB, MYST3, SUZ12P, AEBP2, KDM2B, WHSC1.** KIAA1267, HIST1H2BI, KDM3B, SUV39H2, RBP1, SAP130, HIST1H3E, SMYD2, PADI6, HDAC10, FAM48A, MBD3.
**Cellular responses to stress**	61	**HSPA6, HSPA7, HSPA4L, GPX2, HSPA13, HSPA2, BAG5, HSPA1B, RPS6KA1, RPS6KA2, NPTXR, ETS2, NUP98, ATG16L1, IL6, DNAJB6, DLC1, HSP90AA1, UBE2E2, TXNRD1, ATG9A, RAE1, WDR45L, E2F1, PRKAG2, ACD, CBX2, RAD50, MAPKAPK2, AKT1S1, RPTOR, CCS, HIST1H1A, SOD1, WDR45, NUP62, TXNRD2, GSR, STAT3, ASF1A, TXN2, GML, NECAB3, DYNLL2.** TSC2, GPX1, TNRC6C, TCEB2, TNRC6B, CEBPB, MAP1LC3B, UBC, HSPA1A, EGLN2, PRKAA1, NUP210, ATG3, MAPK14, CBX4, HSPA12B.
**Apoptosis**	25	**TRADD, AKT3, PSMD2, ADD1, TJP2, TNFRSF10D, PSMA7, PSME3, DFFB, PSMB10, LMNA, RIPK1, PSMC4, DIABLO, STK24, DK5RAP2, TRAF2, PSMD11, PTK2.** UNC5A, PSMB9, EPPK1, CASP7, OCLN, BMF.
**Glucose Metabolism**	20	**GAPDHS, FBP2, GLG1, ALDOA, GYG2, PFKFB3, MDH2, GCK, PHKG2, PFKP, HK3, SLC25A10, SLC25A11, PYGM.** UBC, PGAM2, GYS2, PFKL, PGM3, SLC25A1.

The hypermethylated genes are shown in bold letters.

**Table 2:** Cellular pathways and genes with differential DNA methylation in MDA-MB-231 cells at 48h after treatment with resveratrol.

Pathway name	Number of genes	Hypermethylated (bold letters) and hypomethylated genes
**Cell cycle**	113	**HIST1H3F, DKC1, UBE2D1, HIST1H2BG, TOP2A, HSPA2, NUP133, PSMB5, HIST1H2BK, TCP10, G6PC, YWHAG, CCNB1, NUP98, CDC25A, CENPN, MTOR, SYCP3, RNF168, APEX1, HIST1H4E, RAB8A, CEP72, HIST1H4D, OFD1, REC8, FAM175A, MIS12, POLE2, PSMD11, CLASP1, CEP192, CDC6, RAD1, PSMD1, ODF2, BUB1B, POLD1, CEP70, DYNC1H1, RBL1, GMNN, MCM2, PSMC5, FZR1, NEK2, LPIN1, SFN, TUBGCP5, TUBGCP3, CDK5RAP2, HAP1, POM121C, PDS5B, NECAB3, HIST1H2AL.** RCC2, HIST1H2BI, MAD1L1, APITD1, PPP2R2D, PRKCA, PPP2R2A, PIAS4, CSNK2B, HIST1H3D, HIST1H3E, UBC, ANKLE2, TP53, INCENP, HSP90AA1, CCND1, CENPO, PSME3, PSMB10, PSME2, HIST1H4L, CENPP, PPP2R3B, ORC1L, E2F1, CCNL1, ATRIP, BUB3, ACD, HIST2H4B, RAD50, WHSC1, TUBA1C, DIDO1, TEX12, CSNK2A1, PRKAR2B, POLE, CUL1, ANAPC2, APC2, PPP1CA, SMC3, PCNT, TUBGCP2, LPIN2, SKP1, DCTN1, MAPRE1, NUP210, LMNA, TYMS, MCPH1, SYNE2, GINS2, PSMB9.
**Immune System**	112	**GRB2, GBP5, STK4, AP1S3, CARD9, NRG1, KLRD1, SYK, ASB5, SIGLEC15, CLEC4A, HLA-DOB, DUSP1, TRAF6, ASB16, TNFRSF13B, C1QB, RAET1G, PSMD11, CSK, DLG1, FBXO44, NEDD4, ARPC2, DUSP10, VTN, CTSH, CD4, SH3KBP1, UBE2A, ISG15, JAK3, BPI, CD300LF, DYNC1H1, SRMS, MEF2C, STAT6, LGALS9, CTSE, RASGRF2, AGER, PTEN, HLA-DMB, UBE2G2, CRTAM, KLC4, CD200R1L, IL3RA, CD8A, TNFRSF11B, VAV3, KSR1, VAV1, NLRC4, NLRC5.** DLG4, CNKSR1, PRKCA, PYCARD, RPS6KA2, ITGB1, SPTBN5, IL1B, SPTBN4, ACTR3B, GH1, GH2, TNRC6C, CSF2, TNRC6B, TNFRSF6B, EIF4G3, MRC2, EIF4G1, RASGEF1A, CD79B, TRAF2, NFKBIE, ASB1, GZMM, ZAP70, UBE2K, GHR, AKT1S1, RNF135, IFITM1, SEC24C, IFITM2, PRKAR2B, TRAF7, RNF144B, CUL1, ANAPC2, IL17RD, LGALS3, LIF, CANX, CD200R1, IL27RA, MICB, CCL17, SEC61G, FLNB, SKP1, MX1, NUP210, UBA6, FOXO1, PDGFRB, FOXO3, PDGFRA.
**DNA Repair**	80	**FANCA, APEX1, HIST1H2BG, MPG, XRCC3, UBE2A, ISG15, POLD1, PRPF19, POLR2D, INO80, RASA4, KIAA0146, ERCC1, LAMP2, KDM4A, RNF168, HIST1H4E, BAZ1B, HIST1H4D, CSN3, MSH2, ASCC2, FAM175A, HAP1, POLE2, INO80C, MED1, INO80B, EYA3, EYA2, NEIL2, ZBTB32, FANCI, PMS2, MBD4.** GTF2H4, POLM, COPS3, USP10, HIST1H2BI, POLL, APITD1, POLE, PIAS4, XPC, KIAA1530, XPA, UBC, HERC2, ATRIP, POLR2J, TFPT, NFRKB, LIG3, CUL4A, ALKBH3, GPS1, ERCC6, RAD51L3, C1orf86, ERCC2, KDM4B, HIST1H4L, ABL1, RTEL1, PNKP, HIST3H3, C19orf40, UBE2N, DDB2, BACH1, CDS1, SUMO3, DCLRE1A, NTHL1, HIST2H4B, RAD50, WHSC1.
**Signaling by GPCR**	353	**VIPR2, PRKCH, VIPR1, GPR39, F2R, OXT, GNAL, HRH3, TACR2, NRG1, GOLT1A, MCHR1, CRH, TAS1R2, DUSP1, BICD1, NPSR1, CCKAR, PDE4D, PDE4B, RAMP1, PSMD11, RGS4, CSK, RGS7, RHOG, DLG1, OR2C3, DUSP10, GPR4, GPR120, ADRB1, GRM2, CX3CR1, GCGR, OR1F2P, UTS2R, CCBP2, EDN2, JAK3, GPR109A, EDNRB, PTGER1, RASGRF2, TAS2R39, JAK3, OR52W1, IL3RA, OPRK1, ADRB1, ADRB3, CXCR4, BDKRB1, KALRN, CXCR7, GPR84, VAV3, KSR1, FPR3, VAV1, OR51B2, OR52H1, CXCL9, OR8S1, AVP, CXCL5, PSMB5, OR10A3, ABHD6, G6PC, CALCR, PROKR2, GPR84, TIAM1, HTR1D, OR52I1, GALR3, FGF22, CCL22, DHH, RASGRP1, MMP2, GPR68, HBEGF, OR6B3, C5, PIK3R5, CREM, GRM6, GRM8, OR52B6, CCR7, GRPR, AKT1, RASGRF1, CCR9, RASGRF2, SRC, C5AR1, FGFR1, PSMD1, IL8, F2, RASA4, GNRH2, MCHR1, PVR, GNG4, EMR2, GNG8, CCL19, TAAR8, PSMC5, GNA11, GPR4, RAC1, GNAT3, GNA13, RGS10, GRIN1, PRKCQ, RGS13, NET1, OR2A25, PSAP.**DGKZ, DLG4, CNKSR1, DGKQ, PRKCA, ABR, RPS6KA2, DGKI, PTCH2, SHH, PTCH2, DGKD, RGR, OR1L3, LTB4R, SPTBN5, SPTBN4, GNG12, TAS1R1, OR4F6, OR4F5, CSF2, OR4F4, PDE4C, RASGEF1A, RHO, RGS9, GHR, ADM2, PRKAR2B, GRM5, IL17RD, MC5R, PPP1CA, SOS2, CCL17, TIAM2, FGD4, TBXA2R, NPFFR1, HRH1, TRPM2, IL17RD, GRK5, CXCR5, PDE2A, ABR, OR10V1, PDGFRB, PDGFRA, OR2B11, FGD3, OR2A7, FGD4, PROKR1, GLP2R, PLCB2, CD97, KL, SHC2, AGT, WNT9A, ARHGEF16, OR2A4, ARHGEF17, OR51I2, OR2A1, EGFR, AGT, ARHGEF7, HCRTR1, KISS1R, PIK3R1, PIK3R2, PDPK1, MAP2K2, OR6B1, OR6B2, OR9Q2, CX3CL1, PTAFR, ARHGEF4, ARHGEF3, PDGFA, CCL28, OR2A42, CCL27, PLEKHG5, FGF3, FGF4, GAB2, ITPR1, F2RL3, ITPR3, LPAR1, PIK3CG, ADCY7, RASAL2, PPYR1, WNT1, ADCY9, MC1R, CCL17, KLB, GALR2, PNOC, GPR176, FFAR1, FFAR2, S1PR5, DAGLA, CSF2RA, NPBWR2, NPBWR1, GRIN2D, TRPC7, ADRBK1, TAS1R3, SSTR4, GHRL, ADORA3, SSTR2, SSTR3, NRG2, RLN3.
**Chromatin organization**	82	**HIST1H3F, BRD1, HDAC3, HIST1H2BG, SMARCA4, HIST1H2BK, KDM5B, MLL2, EZH2, MLL4, ACTL6B, DNMT3A, MTA3, HCFC1, EATS4, YEATS2, PRMT5, KDM4A, MORF4L2, HIST1H4E, ARID1B, HIST1H4D, TADA2B, C12orf41, HIST1H2AL.** DR1, HIST1H2BI, CHD3, KDM3B, CHD4, MLL, TRRAP, HIST1H3D, KDM5C, SAP130, HIST1H3E, PADI2, PADI6, MTA1, PRDM16, PADI1, TRRAP, KIAA1267, NSD1, KDM4B, HIST1H4L, SMARCD3, EP400, RUVBL2, SUPT3H, DOT1L,SUZ12P, KAT2A, FAM48A, HIST2H4B, WHSC1, GATAD2A, KDM5C, SUV39H2, ASH2L, HIST3H3, NCOR2, HDAC10, JAK2, EHMT1, CSRP2BP, KDM6B, PRMT7, CREBBP, KDM6B, HIST3H3, SETD3, RBP1, SETD1B, SETD7, ING5, SMYD2, MBD3, AEBP2, KDM2B, WDR5.
**Cellular responsesto stress**	106	**MAPKAPK5, MTMR14, UBE2D1, HIST1H2BG, HSPA2, NUP133, HIST1H2BK, CCS, EZH2, HBXIP, IL8, NUP98, TNIK, GPX7, CABIN1, FZR1, MTOR, ASF1A, HIST1H4E, MAP2K3, HIST1H4D, WDR45L, MAPK10, CBX6, POM121C, MINK1, NECAB3, SCMH1, HSPA12A, EPAS1, HIST1H2AL.** HSPA6, HSPA7, HSPA4L, GPX2, HSPA13, GPX1, WIPI2, HIST1H2BI, PC, BAG5, HIST1H3D, MAP1LC3B, HSPA8, HIST1H3E, MAP1LC3A, CDKN1A, UBC, HSPA1A, PRDX2, RPS6KA2, ETS2, NPTXR, ATG16L1, IL6, DNAJB6, DLC1, HSP90AA1, TXNRD1, TSC2, ATG9A, ULK1, HIST1H4L, TNRC6C, EP400, RAE1, TNRC6B, CEBPB, TFDP1, EGLN2, E2F1, PHC2, MAPK14, ATG3, PRKAG2, SUZ12P, CBX4, HIST2H4B, ACD, CBX2, RAD50, AKT1S1, MAPKAPK2, RPTOR, ANAPC11, SOD1, ANAPC2, APC2, HIST3H3, NUP62, WDR45, EHMT1, TXNRD2, GSR, CHMP6, KDM6B, CREBBP, TCEB2, PRKAA1, NUP210, GML, HSPA12B, DYNLL2.
**Apoptosis**	46	**GSN, AKT1, PSMC5, FADD, PSMB5, TICAM1, G6PC, RIPK1, SFN, PSMD1, YWHAG, DIABLO, PRKCQ, CDK5RAP2, KPNB1, PSMD11, NECAB3.** TRADD, PSMB9, AKT3, OCLN, DAPK3, PSMD2, UNC5A, UBC, APC2, ADD1, TJP2, PLEC, TNFRSF10D, DLC1, EPPK1, DFFB, PSME3, PSMB10, PSME2, MNA, PSMC4, TFDP1, STK24, CASP7, E2F1, LMNA, TRAF2, BMF.
**Glucose Metabolism**	28	**GYS1, ENO2, PHKG2, PPP1R3C, PKM2, HK2.** FBP2, PGAM2, GLG1, ALDOA, GYS2, GYG2, PFKP, HK3, PC, HK1, SLC25A1, PFKL, GAPDHS, UBC, PPP2R5D, PFKFB3, MDH2, GCK, SLC25A10, SLC25A11, PGM3, PYGM.

The hypermethylated genes are shown in bold letters.

### Resveratrol alters the DNA methylation marks of oncogenes and tumor suppressor genes

**Fig 5:**
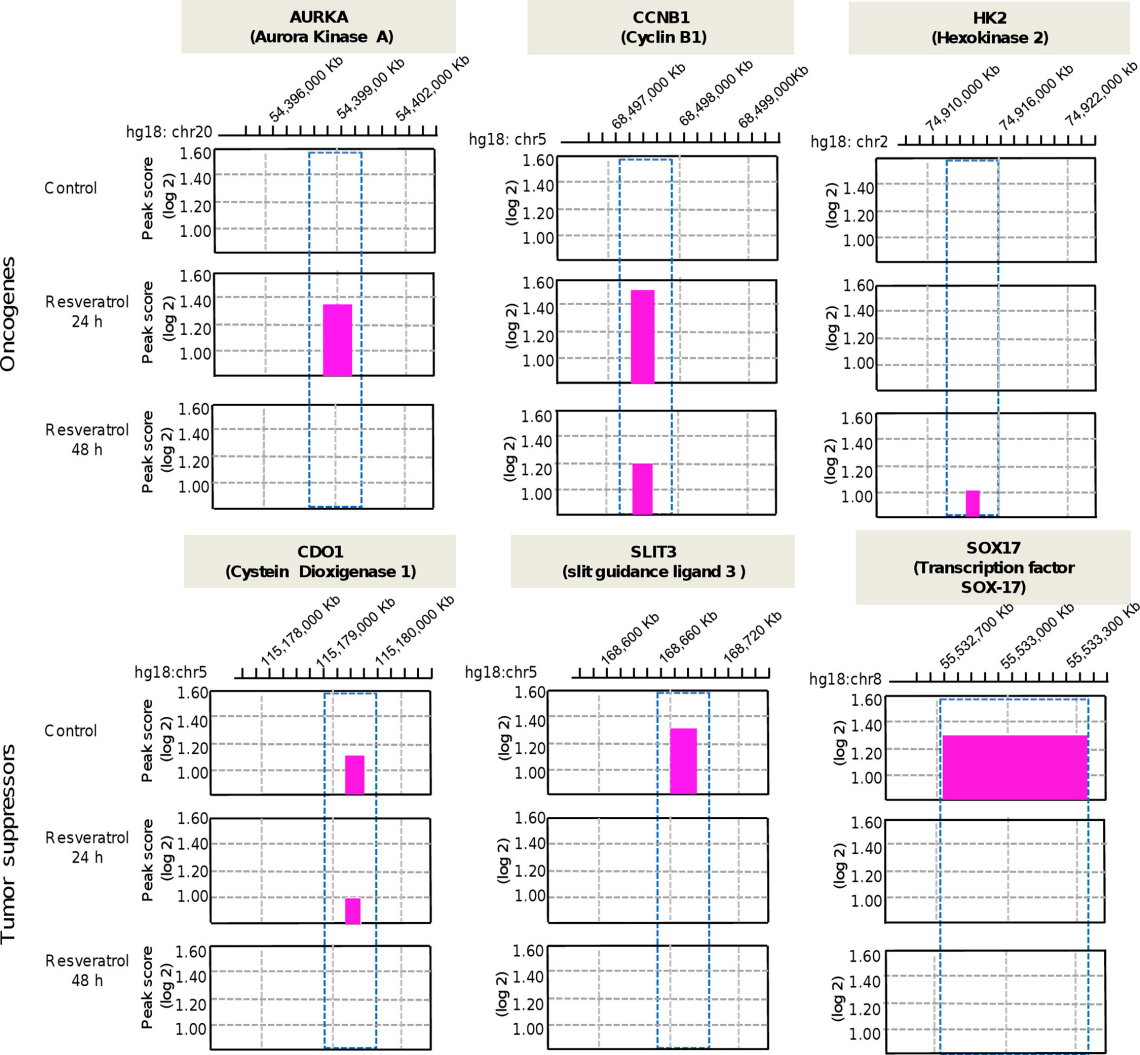
DNA methylation changes in cancer-related genes after resveratrol treatment for 24 h and 48 h. The y-axis indicates the peak value or positive enrichment in IP-based methylation microarray data using a modified ACME algorithm. The x-axis shows the chromosomal location of regions with significant change in the log2 peak value (pink bar) in MDA-MB-231 cells treated with resveratrol (100 μM) and control cells. Blue boxes show the positions in gene promoters with significant changes in DNA methylation.

During the bioinformatic analysis of modulated gene promoters by resveratrol, we observed that several genes that significantly changed its methylation status correspond to key oncogenes and tumor suppressor genes. For example, after 24 h resveratrol intervention 19 tumor suppressor genes changed from hypermethylated to hypomethylated status; these include IGF2R, IFR4, MST1, FOXO3, GNAT1, MST1, MST1R, RPL5, TSC2, WIF1, STK11, TCF3, and DDB2 among others (S4 Table). Meanwhile, after 48 h resveratrol treatment the DNA methylation of 30 tumor suppressor genes such as DICER, TP53, IGFR2, FOX1, FOXO3, GNAT1, NOTCH1, NOTCH3, PAX5, ZMYND10, and WIF1 among others was also decreased (S5 Table). On the other hand, a subset of 20 and 21 hypomethylated oncogenes turned out to hypermethylated after 24 h and 48 h resveratrol treatment, respectively (S6 and S7 Tables). Remarkably, we found that resveratrol exerts early and late changes in DNA methylation of specific genes. Representative examples of these changes, peak scores and chromosomal location of six selected cancer-related genes are shown in Fig 5.

The cell cycle regulators Aurora kinase A (AURKA) and cyclin B1 (CCNB1) that were detected as hypomethylated in non-treated MDA-MB-231 cells, changed to hypermethylated after 24 h resveratrol treatment, and then returned to original low methylation levels after 48 h treatment. In a similar fashion resveratrol induced the hypermethylation of hexokinase 2 (HK2), an oncogene that functions as Warburg effect mediator, only after 48 h treatment. These findings are congruent with our gene expression data recently reported indicating that resveratrol downregulates AURKA and HK2 leading to a cell cycle arrest at phase G1 and inhibition of cell proliferation [17, 18, 19]. Moreover, our aPRIMES approach indicates that resveratrol treatment restores the hypomethylated status of transcription factor SOX-17 (SOX-17), slit guidance ligand 3 (SLIT3), and cysteine dioxygenase type 1 (CDO1), three well known tumor suppressors that are frequently suppressed by hypermethylation in breast cancer [20, 21, 22].

### Integrative analysis of DNA methylation and gene expression

**Fig 6:**
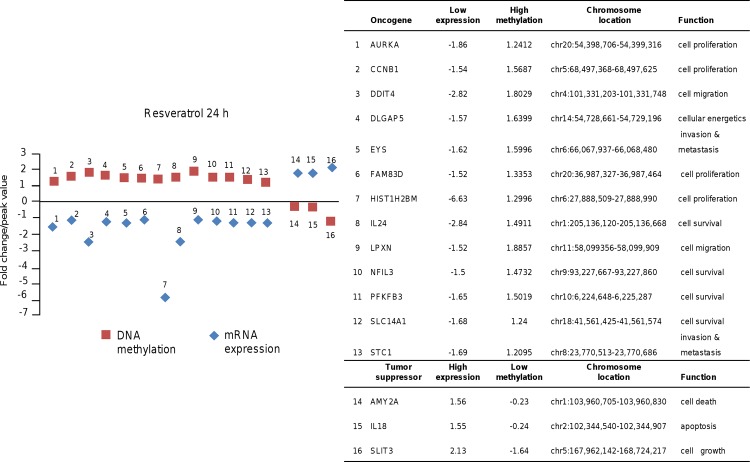
DNA methylation and mRNA expression of oncogenes and tumor suppressor genes in breast cancer cells treated 24 h with resveratrol. Left panel; graphical representation of genes that showed DNA methylation changes after 24 h resveratrol treatment and matched genes with differences (≥1.5) in gene expression. Right panel; oncogenes and tumor suppressor genes with low and high methylation, and mRNA expression values. Chromosomal location of each gene is denoted.

**Fig 7:**
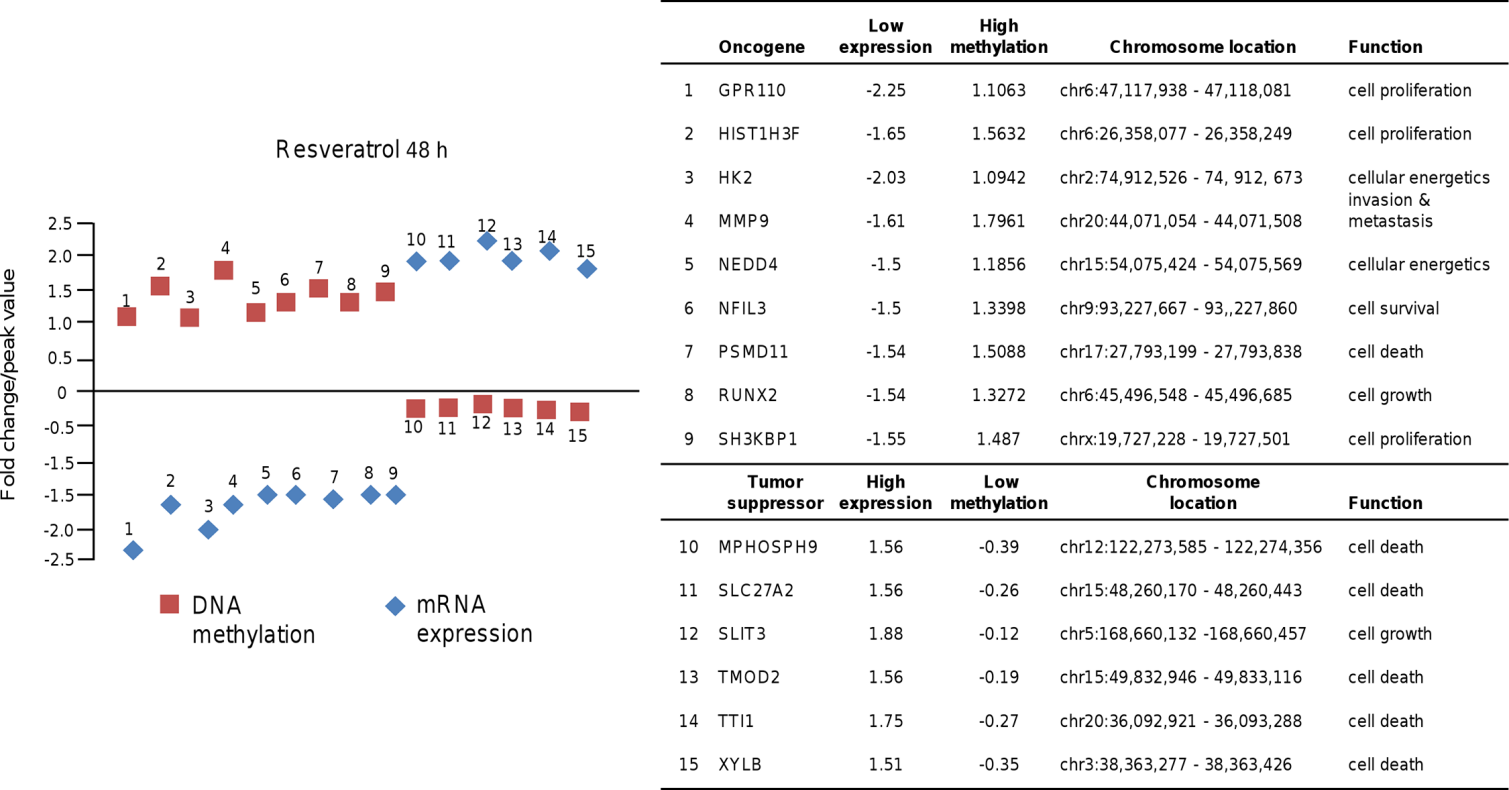
DNA methylation and mRNA expression of oncogenes and tumor suppressors in breast cancer cells treated with 48 h resveratrol. Left panel; graphical representation of genes that showed DNA methylation changes after 48 h resveratrol treatment and matched genes with differences (≥1.5) in gene expression. Right panel; oncogenes and tumor suppressor genes with low and high expression associated to methylation changes. Chromosomal location of each gene is denoted.

To further investigate the biological consequences of altered DNA methylation by resveratrol treatments, we correlated the epigenetic changes with gene expression variations at mRNA level. To achieve this integrative analysis we used the transcriptome data set previously reported in MDA-MB-231 cells treated with resveratrol (100 μM) for 24 h and 48 h [23]. Herein, we selected all CpG *loci* that had a methylation peak >0.9 and an mRNA expression fold change of 1.5 (*p*>0.05) between control and resveratrol treated cells. Using these criteria, we identified 16 and 15 genes modulated at 24 h and 48 h, respectively. In Fig 6 we shown representative data indicating that after 24 h treatment, thirteen selected oncogenes (AURKA, CCNB1, DDIT4, DLGAP5, EYS, FAM83D, HIST1H2BM, IL24, LPXN, NFIL3, PFKFB3, SLC14A1 and STC1) and 3 tumor suppressor gene (AMY2A, IL18 and SLIT3) exhibit a correlation between DNA methylation and mRNA expression levels. A similar behavior was observed for 9 oncogenes and 6 selected tumor suppressor genes after 48 h treatment (Fig 7).

**Fig 8:**
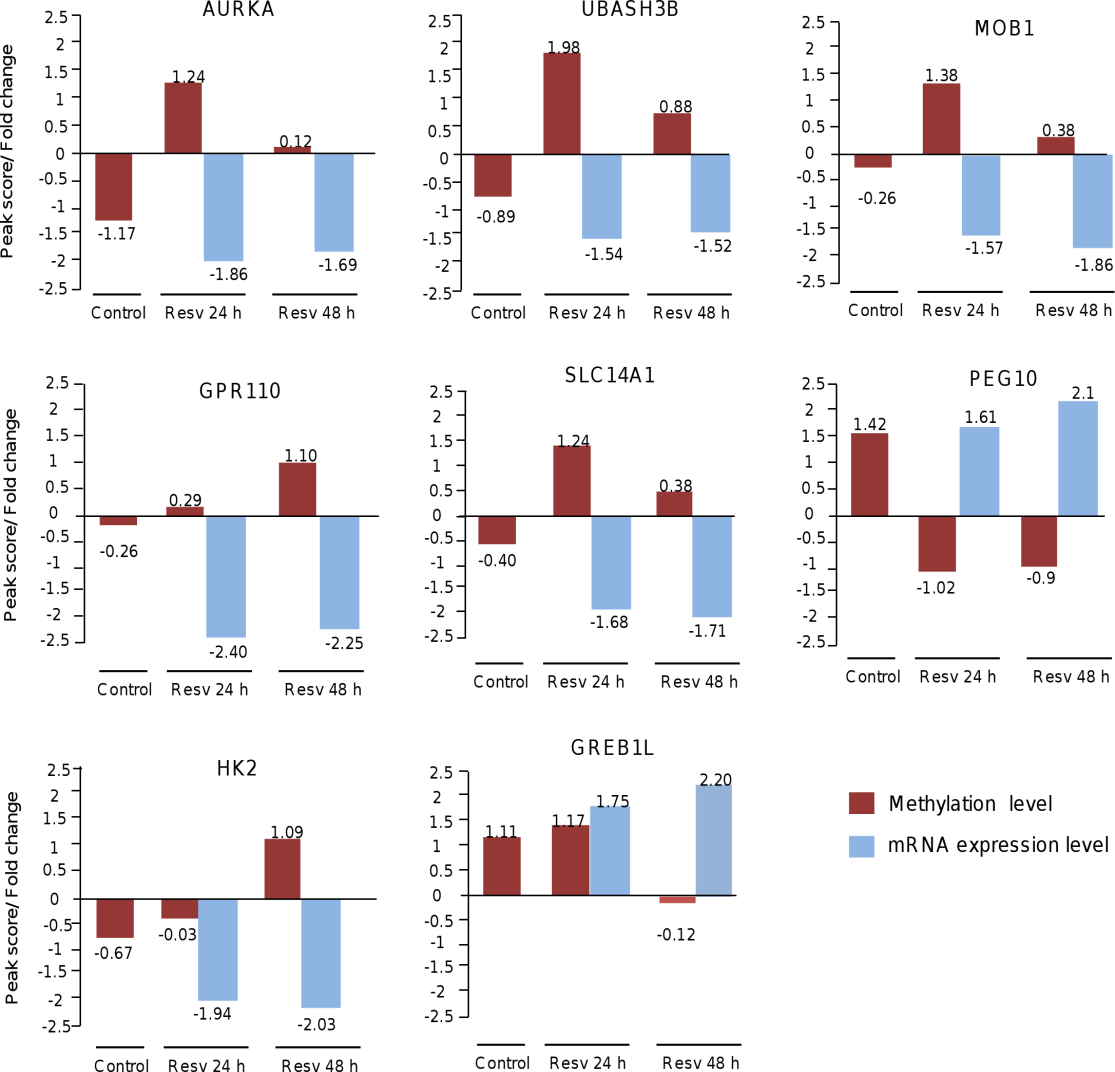
DNA methylation and mRNA expression of selected genes during course of time. Schematic representation of methylation status and mRNA levels of eight modulated genes in MDA-MB-231 cells treated for 24 h and 48 h with resveratrol (100 μM) relative to control non-treated cells. The y-axis represents the methylation levels of gene promoters (peak score) and the differential fold change in mRNA expression relative to control. Black bars indicate the methylation level of specific gene promoters. White bars indicate the gene expression level as obtained from transcriptome analysis [24].

Then we asked if the changes in methylation and gene expression of the selected genes vary during course of time. Our data indicate that after 24 h and 48 h resveratrol treatment 5 out of 8 oncogenes studied (i.e., AURKA, UBASH3B, MOB1, GPR110, and SLC 14A1) showed a high methylation and low mRNA levels (Fig 8). A similar but inverse behavior was observed for PEG10 gene that showed a low methylation level and high mRNA expression level after 24 h and 48 h resveratrol treatment. However, other genes such as HK2 and GREB1L showed intricate methylation/expression patterns after both times of resveratrol treatment suggesting that additional mechanisms are involved in the regulation of gene expression (Fig 8).

## Discussion

Here we provide novel epigenetic data which highlight the relevance of resveratrol on chemoprevention of breast cancer. Breast cancer accounts for 522,000 deaths and was the most frequently diagnosed cancer among women, with 1.7 million cases worldwide in 2012 [25]. Unfortunately tumors frequently recur in patients after first-line treatment; thus alternative therapeutic approaches are needed to overcome increasing drug resistance and improve patient’s survival. The identification of novel epigenetic modulators is an emerging strategy to discover novel nutraceutical drugs with potential chemotherapeutic applications in cancer. In the current study we performed a genome-wide DNA methylation analysis based on promoter DNA microarrays in MDA-MB-231 cells treated with dietary resveratrol which to our best knowledge has not been assessed before in breast cancer. Based upon our data, we showed that resveratrol a polyphenol found in grapes, berries, peanuts, red wine, and plants which exhibits potent and antitumor effects in various types of cancer [26], is a novel modulator of DNA methylation in breast cancer cells. Only few previous reports suggested that resveratrol may exert anti-cancer effects in breast cancer cells through epigenetic mechanisms. For instance, resveratrol inhibits the activity and expression of DNA methyltransferase 1 (DNMT1) in breast cancer cells, which impairs the epigenetic silencing of the BRCA1 tumor suppressor by modulating acetylation of H3K9, and H4, association of mono-methylated-H3K9, DNMT1, and methyl binding domain protein-2 with the promoter of BRCA-1 gene [27]. On the other hand, resveratrol also exhibits epigenetic actions by targeting the chromatin modifier MTA1, histone deacetylases (HDACs) and specific microRNAs [28]. Other study suggests that the histone H2B ubiquitin ligase RNF20, a chromatin modifying enzyme and putative tumor suppressor, is an epigenetic target of resveratrol in breast cancer cells [29]. However, the advances in the knowledge of epigenetic modulation by resveratrol in cancer are still scarce.

In this study we used 100 μM dose of resveratrol to define its impact in global DNA methylation of MDA-MB-231 triple negative breast cancer cells, because we recently analyzed the transcriptome of MDA-MB-231 cells using also 100 μM resveratrol at 24 and 48 h [24], which leads us to correlate the epigenetic changes with gene expression variations at mRNA level and to define how these regulatory mechanisms impacts on expression of specific oncogenes and tumor suppressor genes over time course. We reported that resveratrol induced a decrease in DNA hypermethylation and an increase in DNA hypomethylation of the 22,532 total gene promoters studied here. Importantly, resveratrol did not induce widespread non-specific global methylation, but rather affected only a specific subset of genes suggesting that its DNA methylation-regulatory function was partially independent of DNMT1 inhibition. This effect is desirable because the DNA methylation-modifying agents with little or no effect on global methylation could be more useful and safe in animal and clinical studies, in comparison to the effects caused by pleiotropic drugs targeting the epigenome which may cause a generalized genomic hypomethylation associated with increased genomic instability. A similar and limited effect in DNA methylation was recently reported for curcumin in colorectal cancer cells [30], suggesting that this could be the preferred mechanism of these dietary compounds to modulate the methylome.

Interestingly, resveratrol induced changes in promoter methylation of oncogenes and tumor suppressor genes associated to cellular pathways frequently deregulated in cancer. For instance AURKA, CCNB1, and HK2 oncogenes, among others, changed from hypomethylated to hypermethylated status after resveratrol treatment in a time dependent manner. AURKA and CCNB1 are oncogenes overexpressed in many types of cancer and they are involved in progression of the cell cycle, and positively correlated with tumorigenesis, metastasis and chemotherapy resistance [31, 32]. AURKA overexpression has been associated with aneuploidy, and is a good marker of tumor progression and prognosis. Its deregulation may induces chromosomal instability in several malignancies including breast, colon, pancreas, ovaries, bladder, liver and gastric cancers, whereas CCNB1 (also known as Cyclin B1) belongs to the highly conserved cyclin family and is significantly overexpressed in various cancer types [32]. In addition, resveratrol treatment induces late hypermethylation in the promoter region of hexokinase 2 (HK2), an important oncogene involved in maintenance of glycolysis needed to sustain exacerbated cell proliferation and growth of tumor cells [33]. In agreement, previous studies have indicated that resveratrol downregulates HK2 inducing apoptosis in hepatocellular carcinoma; however the involvement of an epigenetic regulatory event was not described [34]. Congruently, we recently showed that resveratrol suppresses cell cycle by downregulating AURKA and CCNB1 at protein and mRNA level, and also impairs cell proliferation, at least in part, by a decrease of HK2 protein levels [24, and our unpublished data].

Our aPRIMES approach indicates that resveratrol was able to restore the hypomethylated condition of several tumor suppressors. For instance, transcription factor SOX17 (SOX17), slit guidance ligand 3 (SLIT3), and cysteine dioxygenase type 1 (CDO1) that are frequently suppressed by hypermethylation in breast cancer [35, 36], turned hypomethylated after resveratrol treatment. SOX17, a high-mobility group box transcription factor, is a key regulator of development and a negative regulation factor of β-catenin/TCF transcription activity in the Wnt/β-catenin pathway. Hypermethylation of SOX17 promoter was correlated with poor prognosis in esophageal and hepatocellular carcinoma, as well as in colorectal and gastric cancer among other cancers [36]. In addition, SLIT3 interacts with Robo4 to induce tumor angiogenesis, SLIT3 is a glycoprotein that guide axonal development during embryogenesis and cell migration that is frequently hypermethylated and silenced in lung, breast, colorectal and glioma cell lines and primary tumors [37]. CDO1 is a metalloenzyme involved in conversion of the cysteine to cysteine sulfinic acid, while it may promote apoptosis by increasing reactive oxygen species through suppression of glutathione generation. CDO1 plays a tumor suppressive role in human carcinogenesis and is frequently inactivated by promoter methylation in breast, esophagus, lung, bladder, gastric and colorectal tumors [38]. These data suggested that AURKA, CCNB1, HK2, SOX17, SLIT3 and CDO1 might represent novel potential targets for epigenetic therapy in breast cancer. In agreement with a recent report here we identified hypomethylated oncogenes (HK2, FGFR4, DNMT3A) and methylated tumor suppressor genes (CDO1, SLIT3, and SOX17), which were previously identified as candidate genes that comprise part of the emerging “cancer methylome” from 22 cancer cell lines derived from several cancer types as lung, breast, colon, liver, skin, prostate, and cervical cancer, among others [20–22].

On the other hand, we found a good correlation between DNA methylation and gene expression changes in a specific set of cancer-related genes that further highlighted the biological significance of resveratrol-induced methylation alterations in breast cancer cells. These concordant changes in DNA methylation and gene expression were found in oncogenes (AURKA, CCNB1, DDIT4, DLGAP5, EYS, FAM83D, HIST1H2BM, IL24, LPXN, NFIL3, PFKFB3, SLC14A1, STC1, GPR110, HIST1H3F, HK2, MMP9, NFIL3, PSMD11, RUNX2, SH3KBP1) and tumor suppressor genes (AMY2A, IL18, SLIT3, MPHOSPH9, SLC27A2, TMOD2, TTI1 and XYLB). In contrast, other genes such as PEG10 showed an inverse correlation between promoter DNA methylation and gene expression suggesting that additional mechanisms of gene regulation are operating in response to resveratrol. Finally, although our study demonstrates a systematic and compelling effect of resveratrol on DNA methylation in breast cancer cells, data requires further animal and human studies in order to validate *in vivo* these results. Although speculative, our findings permit us to propose that resveratrol may modulate gene expression and exert anti-proliferative activities based on its ability to modify the DNA methylation of well-known cancer genes suggesting that it may be useful as a novel epigenetic therapeutic tool. Additional in vivo studies are needed to evaluate the precise molecular mechanisms of resveratrol-mediated methylation changes and estimate the potential of resveratrol for inducing epigenetic modulations in preclinical models of breast cancer.

## Supporting Information

S1 Table Methylation raw data for MDA-MB-231 control cells. (XLS)

S2 Table Methylation raw data for MDA-MB-231 cells treated with resveratrol (100 uM) for 24 h. (XLS)

S3 Table Methylation raw data for MDA-MB-231 cells treated with resveratrol (100 uM) for 48 h. (XLS)

S4 Table Tumor suppressor genes that change from hypermethylated to hypomethylated status in MDA-MB-231 breast cancer cells treated with resveratrol (100 μM) at 24 h. (DOC)

S5 Table Tumor suppressor genes that change from hypermethylated to hypomethylated status in MDA-MB-231 breast cancer cells treated with resveratrol (100 μM) at 48 h. (DOC)

S6 Table Oncogenes that change from hypomethylated to hypermethylated status in MDA-MB-231 breast cancer cells treated with resveratrol (100 μM) at 24 h. (DOC)

S7 Table Oncogenes that change from hypomethylated to hypermethylated status in MDA-MB-231 breast cancer cells treated with resveratrol (100 μM) at 48 h. (DOC)
