# Supplementary material for: Methylation Landscape of Human Breast Cancer Cells in Response to Dietary Compound Resveratrol
Source: PLoS One. 2016 Jun 29;11(6):e0157866. doi: 10.1371/journal.pone.0157866 (PMC4927060; doi:10.1371/journal.pone.0157866)
Supplement: S5 Table — (DOC) [file pone.0157866.s005.doc]

**Supplementary table 5.** Tumor suppressor genes that change from hypermethylated to hypomethylated status in MDA-MB-231 breast cancer cells treated with resveratrol (100 µM) at 48 h.

| Gen ID | Official Symbol | Log 2 value | Chromosomal location of hypomethylated region |
| --- | --- | --- | --- |
| 84448  79026  55966  1026  863  1496  1643  23405  201163  2308  2309  2779  3482  3662  3977  4221  4485  4486  4627  4851  4854  5079  6125  6794  6929  7157  7249  11197  51364 | ABLIM2  AHNAK  AJAP1  CDKN1A  CBFA2T3  CTNNA2  DDB2  DICER1  FLCN  FOXO1  FOXO3  GNAT1  IGF2R  IRF4  LIFR  MEN1  MST1  MST1R  MYH9  NOTCH1  NOTCH3  PAX5  RPL5  STK11  TCF3  TP53  TSC2  WIF1  ZMYND10 | -1.52  -1.02  -1.18  -1.26  -1.18  -1.22  -1.32  -1.08  -1.57  -1.18  -2.09  -1.54  -1.47  -1.02  -1.25  -1.29  -2.14  -1.06  -1.18  -1.24  -1.08  -1.08  -1.19  -1.17  -1.27  -2.0  -1.31  -1.06  -1.01 | chr4:8,213,153-8,213,706  chr11:61,968,630-61,968,791  chr1:4,612,731-4,612-888  chr6:36,759,633-36,759,904  chr16:87,570,692-87,570,959  chr2:80,403,073-80,403,510  chr11:47,190,996-47,191,347  chr14:94,693,252-94,693,411  chr17:17,065,411-17,065,938  chr13:40,137,800-40,137,961  chr6:109,091,254-109,091,979  chr3:50,206,159-50,206,606  chr6:160,432,081-160,434,242  chr6:338,182-338,411  chr5:38,633,313-38,633,476  chr11:64,329,003-64,329,332  chr3:49,700,364-49,701,197  chr3:49,95,339-49,915,950  chr22:35,048,714-35,048,851  chr9:138,514,925-138,515,374  chr19:15,173,044-15,173,193  chr9:37,024,116-37024,251  chr1:93,075,648-93,075,810  chr19:1,177,504-1,177,639  chr19:1,576,326-1,576,663  chr17:7,518,950-7,519,377  chr16:2,074,203-2,074,846  chr12:63,801,692-63,801,823  chr3:50,354,891-50,355,018 |
