# Supplementary material for: Methylation Landscape of Human Breast Cancer Cells in Response to Dietary Compound Resveratrol
Source: PLoS One. 2016 Jun 29;11(6):e0157866. doi: 10.1371/journal.pone.0157866 (PMC4927060; doi:10.1371/journal.pone.0157866)
Supplement: S6 Table — (DOC) [file pone.0157866.s006.doc]

**Supplementary table 6.** Oncogenes that change from hypomethylated to hypermethylated status in MDA-MB-231 breast cancer cells treated with resveratrol (100 µM) at 24 h.

| Gen ID | Official Symbol | Log 2 value | Chromosomal location of hypermethylated region |
| --- | --- | --- | --- |
| 6790  207  170961  284  8312  657  776  23261  868  8661  2261  2534  3660  4436  4853  4914  9891  861  6657  6774 | AURKA  AKT1  ANKRD24  ANGPT1  AXIN1  BMPR1A  CACNA1D  CAMTA1  CBLB  EIF3A  FGFR3  FYN  IRF2  MSH2  NOTCH2  NTRK1  NUAK1  RUNX1  SOX2  STAT3 | 1.24  1.30  1.26  1.50  1.09  1.30  1.55  1.27  1.46  1.40  1.13  1.14  1.68  1.26  1.27  1.40  1.65  1.65  1.57  1.77 | chr20:54,398,706-54,399,316  chr14:104,311,019,-104,311,666  chr19:4,168,448-4,168,593  chr8:108,580,328-108,580,686  chr16:314,898,-315,137  chr10:88,506,380-88,506,517  chr3:53,501,943-53,502,690  chr1:6,767,756-6,767,928  chr3:107,070,391-107,070,540  chr10:120,832,156-120,832,708  chr4:1,773,498-1,773,743  chr6:112,147,753-112,147,922  chr4:185,614,347-185,615,005  chr2:47,482,737-47,483,085  chr1:120,414,701-120,414,868  chr1:155,051,999-155,052,126  chr12:105,057,349-105,058,799  chr21:35,334,957-35,344,846  chr3:182,913,050-182,913,777  chr17:37,795,422-37,796,071 |
