# Supplementary material for: Methylation Landscape of Human Breast Cancer Cells in Response to Dietary Compound Resveratrol
Source: PLoS One. 2016 Jun 29;11(6):e0157866. doi: 10.1371/journal.pone.0157866 (PMC4927060; doi:10.1371/journal.pone.0157866)
Supplement: S7 Table — (DOC) [file pone.0157866.s007.doc]

**Supplementary data 7**. Oncogenes that change from hypomethylated to hypermethylated status in MDA-MB-231 breast cancer cells treated with resveratrol (100 µM) at 48 h.

| Gen ID | Official Symbol | Log 2 value | Chromosomal location of hypermethylated region |
| --- | --- | --- | --- |
| 207  8312  8019  701  776  23261  8661  2261  2260  2885  3099  4436  4914  9891  861  6657  6774  7048  200424  51592  7227 | AKT1  AXIN1  BRD3  BUB1B  CACNA1D  CAMTA1  EIF3A  FGFR3  FGFR1  GRB2  HK2  MSH2  NTRK1  NUAK1  RUNX1  SOX2  STAT3  TGFBR2  TET3  TRIM33  TRPS1 | 1.30  1.37  1.38  1.10  1.55  1.29  1.28  1.43  1.34  1.41  1.09  1.36  1.36  1.34  1.10  1.57  1.77  1.03  1.21  1.03  1.06 | chr14:104,311,019,-104,311,666  chr16:287,795-288,150  chr9:135,904,041-135,904,714  chr15:38,238,742-38,239,015  chr3:53,501,943-53,502,690  chr1:6,767,756-6,767,928  chr10:120,832,250-120,832,613  chr4:1,773,498-1,773,647  chr8:38,445,848-38,446,597  chr17:70,915,297-70,915,833  chr2:74,912,526-74,912,673  chr2:47,482,647-47,482,947  chr1:155,051,675-155,052,438  chr12:105,058,438-105,058,799  chr21:35,334,957-35,335,196  chr3:182,913,050-182,913,777  chr17:37,795,422-37,796,071  chr3:30,620,962-30,621,211  chr2:74,124,963-74,125,126  chr1:114,857,057-114,857,196  chr8:116,748,864-116,749,115 |
